# The Plasminogen–Activator Plasmin System in Physiological and Pathophysiological Angiogenesis

**DOI:** 10.3390/ijms23010337

**Published:** 2021-12-29

**Authors:** Asmaa Anwar Ismail, Baraah Tariq Shaker, Khalid Bajou

**Affiliations:** 1 Department of Applied Biology, College of Sciences, University of Sharjah, Sharjah 27272, United Arab Emirates; U17102151@sharjah.ac.ae (A.A.I.); U17105703@sharjah.ac.ae (B.T.S.); 2 Human Genetics & Stem Cells Research Group, Research Institute of Sciences & Engineering, University of Sharjah, Sharjah 27272, United Arab Emirates

**Keywords:** angiogenesis, plasminogen-activator inhibitor-1 (PAI-1), plasmin, endothelial cells, vascular endothelial growth factor (VEGF), urokinase-plasminogen activator (uPA)

## Abstract

Angiogenesis is a process associated with the migration and proliferation of endothelial cells (EC) to form new blood vessels. It is involved in various physiological and pathophysiological conditions and is controlled by a wide range of proangiogenic and antiangiogenic molecules. The plasminogen activator–plasmin system plays a major role in the extracellular matrix remodeling process necessary for angiogenesis. Urokinase/tissue-type plasminogen activators (uPA/tPA) convert plasminogen into the active enzyme plasmin, which in turn activates matrix metalloproteinases and degrades the extracellular matrix releasing growth factors and proangiogenic molecules such as the vascular endothelial growth factor (VEGF-A). The plasminogen activator inhibitor-1 (PAI-1) is the main inhibitor of uPA and tPA, thereby an inhibitor of pericellular proteolysis and intravascular fibrinolysis, respectively. Paradoxically, PAI-1, which is expressed by EC during angiogenesis, is elevated in several cancers and is found to promote angiogenesis by regulating plasmin-mediated proteolysis and by promoting cellular migration through vitronectin. The urokinase-type plasminogen activator receptor (uPAR) also induces EC cellular migration during angiogenesis via interacting with signaling partners. Understanding the molecular functions of the plasminogen activator plasmin system and targeting angiogenesis via blocking serine proteases or their interactions with other molecules is one of the major therapeutic strategies scientists have been attracted to in controlling tumor growth and other pathological conditions characterized by neovascularization.

## 1. Introduction

During embryogenesis, the first organ system to emerge in complex multicellular organisms is the cardiovascular system, which provides the embryo with proper vasculature for the delivery of oxygen and nutrients and disposal of metabolic wastes. Mesodermal stem cells give rise to hemangioblasts, which in turn give rise to angioblasts with the potential to develop into endothelial cells (EC) [1]. In early embryonic development, angioblasts from the lateral mesoderm drift to the midline towards vascular endothelial growth factor-A (VEGF-A) stimulus forming a primordial vascular plexus [2]. The process through which endothelial precursor cells differentiate into mature EC and establish a nascent vascular network is known as vasculogenesis. EC sequentially makes up the laminal surface of the vascular system in vertebrates. The formation of vasculature from preexistent EC is a process known as angiogenesis [3]. Angiogenesis is first observed when EC from the plexus, in response to indigenous angiogenic stimuli and hypoxia, raid adjacent vascular-free areas and form new intricate capillary networks [4]. It is a tightly regulated physiological process not only essential for embryogenesis, but also for development, growth, wound healing [5], inflammation and immune responses [6]. In the reproductive organs of females, angiogenesis is essential for processes such as ovulation, corpus luteum development [6], menstruation, and embryo implantation [7]. It is also associated with various pathophysiological conditions like diabetes, cancer, inflammatory disorders [8], and retinal neovascularization [9]. Angiogenesis can be divided into two phases known as the activation and resolution phases. During the activation phase, vessels are permeabilized and fibrin is deposited [10]. The next step includes the hydrolysis of the extracellular matrix (ECM) and basement membrane by proteases. EC then migrate and proliferate, forming branches in the lumen called primary sprouts, and new lumen is subsequently formed at the migrating tip or branch. As the resolution phase commences, proliferation and migration of EC is terminated, the basement membrane is reconstructed, junctions are restored and pericytes are recruited to cover EC and form mature stable vessels [5,11,12,13]. Vessel homeostasis and quiescence are achieved by a balance between angiogenic stimulators such as basic fibroblast growth factor (bFGF), VEGF, and chemokines and between angiogenic inhibitors like angiostatin, interferons, and endostatin [11]. In cancer, an imbalance between these factors causes a switch to a proangiogenic state leading to increased vessel formation and tumor progression [14]. In 1971, Judah Folkman demonstrated that angiogenesis is fundamental for tumor growth and that tumors are not capable of growing more than 1–2 mm^3^ in diameter in angiogenesis-free environments [15].

VEGF-A, which is the most powerful angiogenic activator and mitogen for vascular EC, is overexpressed in response to hypoxia via the activation of the hypoxia-inducible factor-1α (HIF-1α). VEGF-A, secreted by hypoxic tumor cells, binds to tyrosine kinase vascular endothelial growth factor receptor 2 (VEGFR2) on neighboring EC, thereby activating a series of signaling pathways downstream responsible for the proliferation and migration of EC. In 1989, Folkman and colleagues used transgenic Rip1Tag2 mice overexpressing certain oncogenes as a model to show the development from a hyperplastic to a neoplastic tumor and confirmed that VEGF-A is the principal factor in angiogenesis and in EC’s migration and proliferation [16]. VEGF-activated EC produces matrix metalloproteinases (MMPs) with the ability to degrade the surrounding ECM [17,18,19]. Consequently, cytokines and growth factors sequestered within the ECM are released. Together with VEGF-A, released growth factors and cytokines activate signaling pathways responsible for EC’s proliferation and migration towards the source of proangiogenic molecules [13,20,21]. A plethora of in vivo studies confirmed that the deficiency in MMPs resulted in reduced angiogenesis or, in some cases, suspended angiogenesis [21,22]. In vivo, mice deficient in both MMP-2 and MMP-9 showed significant impairment of angiogenesis [23]. Beside MMPs, four other proteinase families exist, including cysteine, threonine, and aspartic and serine proteases, which are categorized based on the catalytic activity of the chemical group attached [24].

Serine proteases, specifically the plasminogen activator plasmin system, are vital players in the ECM remodeling process necessary for angiogenesis. The plasminogen activator–plasmin system is comprised of the active protease plasmin and its proenzyme plasminogen (Plg), urokinase/tissue-type plasminogen activators (uPA/tPA), urokinase-type plasminogen activator receptor (uPAR), plasminogen-activator inhibitor-1 (PAI-1) and PAI-2 [25]. Plasmin is a potent serine protease with an extensive range of substrates like vitronectin, thrombospondin, laminins, and fibrin. Plasminogen is a zymogen produced by hepatic cells and is cleaved at peptides Arg561-Val562 by uPA and tPA to form active plasmin [26]. tPA and uPA are also serine proteases with plasminogen as their major substrate [10]. PAI-1 is a serine protease inhibitor that inhibits plasminogen activators from converting plasminogen into plasmin [21]. In this review article, the plasminogen activator–plasmin system in physiological and pathophysiological angiogenesis will be discussed in greater detail and the mechanisms used by members of this system to induce tumor angiogenesis will be reviewed thoroughly.

## 2. Plasminogen Activator–Plasmin System

### 2.1. tPA

tPA is a fibrin-dependent enzyme primarily involved in dissolving blood clots [24]. Intravascularly, the serine protease thrombin converts soluble fibrinogen into insoluble fibrin which is the chief constituent of a thrombus. Fibrin is then degraded with the help of plasmin yielding fibrin degradation products (FDPs) through a process known as fibrinolysis. In normal physiological conditions, a balance between coagulation and fibrinolysis is maintained intravascularly by maintaining a balance between plasminogen activators (tPA/uPA) and inhibitors (PAI-1/α_2_-antiplasmin). If fibrinolysis prevails, intravascular bleeding develops, whereas thrombosis develops when clotting prevails [27,28]. tPA is produced by keratinocytes, endothelial, and brain cells as a single chained 70 kDa glycoprotein with low proteolytic activity and a plasma concentration of (5–10 ug/L) [29]. The proenzyme undergoes cleavage of the peptide bond between Arg^275^-Ile^276^ by plasmin or kallikrein to form double-chained mature tPA [26,30].

**Figure 1:**
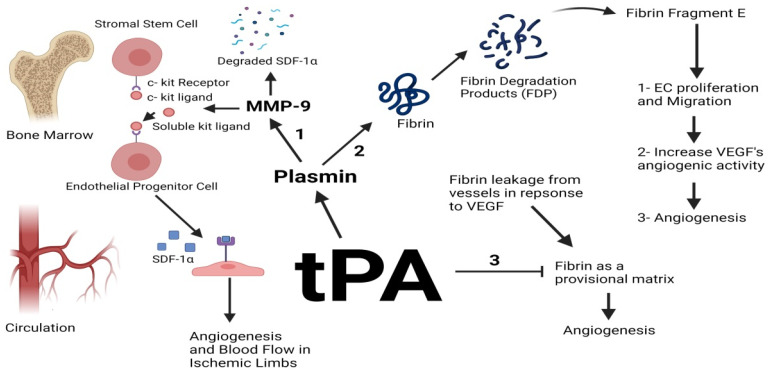
Tissue-type plasminogen activator (tPA) induces angiogenesis by various mechanisms. (1) Plasmin-activated matrix metalloproteinase-9 (MMP-9) cleaves the membrane-bound c-kit ligand on stromal stem cells resulting in the release of soluble kit ligands that bind to their receptors on endothelial progenitor cells, thereby stimulating endothelial cell’s (EC) migration to the circulation. MMP-9 also degrades stromal cell-derived factor 1-α in the bone marrow, thereby accumulating it in the circulation where it also augments angiogenesis and blood flow. (2) Fibrin fragment E generated as a result of fibrin degradation by plasmin induces EC proliferation and migration and increases vascular endothelial growth factor’s (VEGF) angiogenic activity, thereby stimulating angiogenesis. (3) VEGF induces vascular permeability and the leakage of fibrin from vessels which serves as a provisional matrix for EC proliferation, migration, and tube formation. tPA-activated plasmin causes the degradation of fibrin, thereby inhibiting angiogenesis.

In a chorioretinal disease model, uPA^−/−^, tPA^−/−^ and Plg^−/−^ mice exhibited a significant decrease in choroidal neovascularization compared to the wild type confirming the association of these molecules in pathological angiogenesis such as in choroidal neovascularization (CNV) [31]. Little is known about the role of tPA in tumor angiogenesis; however, ex vivo cultures of aortic rings attained from tPA^−/−^ mice and grown on Matrigel and collagen-I lattices displayed a total failure in capillary sprouting similar to that seen in vessels retrieved from Plg deficient mice confirming that tPA and Plg are essential for angiogenesis in this model. MMP-9 activity was also undetectable in explants retrieved from tPA and Plg deficient mice suggesting that tPA induces angiogenesis via the plasmin-mediated activation of MMPs [32]. Various in vivo studies in critical limb ischemia (CLI) showed a correlation between tPA and angiogenesis and proposed mechanisms to explain this association. tPA was found to upregulate the activity of MMP-9 in the bone marrow; MMP-9 hence cleaves the membrane-bound kit ligand on stromal stem cells forming soluble kit ligands. The soluble ligands consequently bind to the c kit receptor on endothelial progenitor cells (EPCs), initiating a signaling cascade downstream vital for the differentiation and mobilization of EPCs from the bone marrow to the circulation. The upregulation and activation of MMP-9 by tPA causes MMP-9 to degrade stromal cell-derived factor 1-α (SDF1-α), which is a potent proangiogenic factor in the bone marrow, thus increasing its concentrations in the circulation and decreasing it in the bone marrow, thereby augmenting angiogenesis and blood flow in ischemic limbs [33,34,35] (Figure 1). 

Another mechanism by which tPA induces angiogenesis is explained through its ability to activate plasmin using fibrin as a cofactor. VEGF expressed by tumor cells induces vascular permeability and fibrin leakage from plasma. tPA then uses fibrin as a cofactor to activate plasmin which in turn degrades fibrin into FDPs, which include fibrin fragment E. This specific fragment has been found to stimulate EC proliferation and migration and increase VEGF’s proangiogenic activity [36,37,38]. Fibrin fragment E caused neovascularization in a chick chorioallantoic (CAM) assay and encouraged the outgrowth of smooth muscle cells from rabbit aortic explants in vitro [39]. In another model, treatment of induced angiogenesis in rabbit’s cornea showed an antiangiogenic effect of tPA, but this effect was not seen at the level of HUVEC cell growth and viability. This can be explained by the fact that fibrin deposition during the process of angiogenesis constitutes a scaffold used by EC to form new vessels. The authors claimed that exogenous tPA can inhibit the formation of the fibrin scaffold and block the formation of new vessels and suggested that intravitreal injection of tPA might prevent retinal angiogenesis [40]. In a retinal neovascularization model, a small peptide generated from kringle 2 domain (His_65_- Tyr_76_) of tPA attained antiangiogenic effects on EC in vitro and in vivo. In VEGF-induced EC, the peptide inhibited EC proliferation, tube formation, and migration dose-dependently making this peptide a potential drug for treating retinal angiogenesis [41].

### 2.2. uPA

Single-chained uPA (ScuPA) is produced by fibroblasts [42], EC and keratinocytes and is synthesized as a single chained glycoprotein 55 kDa in size. Its plasma concentration is (5–10 ug/L), and it is converted into its two-chained active form via the cleavage of the peptide bond between Lys^158^-Ile^159^ by kallikrein, plasmin, cathepsin, or coagulation factor XIIa [29]. uPA is comprised of two domains known as the N terminal growth factor domain, which is the binding site for uPAR and the C terminal catalytic domain [24]. Unlike tPA, uPA is fibrin-independent and is predominantly involved in ECM remodeling and pericellular proteolysis [43]. uPA is activated upon binding to its specific urokinase-type plasminogen activator receptor, which is a glycosyl-phosphatidyl inositol-linked (GPI) cell surface receptor [44]. ScuPA is an inert precursor of uPA that binds with great affinity to uPAR, thereby activating uPA and stimulating the production of plasmin. uPA plays vital roles in physiological angiogenesis, such as in wound healing [45], migration of neural cells, and vascular remodeling [46]. Using in situ hybridization, a study confirmed that uPA mRNA was detected during ovarian follicle’s neovascularization and throughout the corpus luteum vessel’s development, confirming the roles played by uPA in physiological angiogenesis [47]. In pathological angiogenesis and in a myocardial infarction (MI) model, uPA^−/−^ mice displayed inhibition of angiogenesis post-MI [48]. uPA^−/−^ and uPAR^−/−^ mice subcutaneously injected with murine prostate cancer cells displayed a significant decrease in the volume of the tumor and vessels density in both models compared to the wild-type control. The reduction of tumor angiogenesis was associated with a substantial decrease in the number of infiltrating macrophages into the site of the tumor, which are known for their ability to promote angiogenesis and tumor invasion. The contribution of uPA and uPAR to the tumor microenvironment is thus crucial for angiogenesis and tumor progression [49]. In a human glioblastoma model, downregulating uPA using antisense-uPA instigated a decrease in cellular invasion and phosphatidylinositol 3-kinase (PI3K)/Akt phosphorylation. Further analysis in the same model confirmed that uPA stimulates angiogenesis through PI3K/Akt activation, which has been implicated previously in actin reorganization and migration/invasion of EC [50]. However, no significant differences in tumor growth and angiogenesis were observed when malignant keratinocytes were orthotopically implanted in mice deficient in uPA, and it was observed that uPA deficiency was associated with an increase in tPA activity at the tumor site. Using the same skin tumor model, the levels of uPA increased in tumors implanted in tPA deficient mice. Altogether, the data showed a compensation mechanism between tPA and uPA in the keratinocyte tumor model [51] (Table 1). In vitro studies also confirmed that uPA is highly expressed in various types of cancer cells like ovarian cancer cells [52] and in surrounding stromal cells [53]. Immunohistochemical staining of skin biopsies from basal cell carcinoma patients displayed elevated expression of uPA and uPAR compared to normal skin biopsies and suggested involvement in psoriatic angiogenesis [54]. In breast cancer, high levels of uPA are correlated with poor clinical outcomes and are considered as strong prognostic factors [55]. Overall, the previous data points out the importance of plasminogen activation by uPA in the process of angiogenesis.

**Figure 2:**
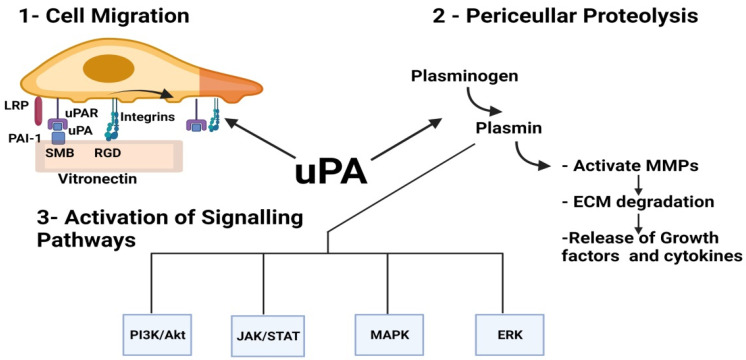
uPA induces angiogenesis by interacting with cell-matrix proteins (vitronectin and integrins), inducing plasmin-mediated pericellular proteolysis and activating signaling pathways. (1) The binding site of plasminogen activator inhibitor-1 (PAI-1) and urokinase-type plasminogen activator receptor (uPAR) to vitronectin is on the somatomedin B (SMB) domain integrins, however, bind to vitronectin through the RGD domain. As PAI-1 binds to uPA, the binding of uPAR and integrins to vitronectin will be inhibited, thereby releasing the cell from general matrixes and recycling uPAR and integrins to the leading edge of the cell via the clearance receptor lipoprotein receptor-related protein (LRP). (2) uPA-activated plasmin activates MMPs and induces pericellular proteolysis and the release of sequestered proangiogenic cytokines and growth factors. (3) Plasmin induces the activation of various pathways involved in tumor proliferation and migration.

It has been described in the literature that uPA stimulates angiogenesis in various ways. Assorted studies presented a correlation between uPA and VEGF-mediated vascular sprouting in which nuclear uPA binds to haematopoietically-expressed homeobox protein (HHEX) transcription factor, thereby upregulating the expression of VEGFR 1 and 2 [56]. uPA also catalyzes the conversion of plasminogen into plasmin which plays various angiogenic roles such as activating proMMPs, liberating growth factors sequestered within the ECM and activating several signaling pathways responsible for cellular proliferation and migration such as the Janus kinase/signal transducer and activator of transcription (JAK/STAT), p38 mitogen activated protein kinase (MAPK) pathways and extracellular signal-regulated protein kinase (ERK)1/2 [26]. For example, a 135 amino acid amino terminal fragment (ATF) of uPA capable of binding to uPAR but lacking proteinase activity was able to activate ERK pathway in MCF-7 cells, promoting their migration in vitro [57]. uPA-activated plasmin is also responsible for the activation of transforming growth factor-β (TGF-β), which stimulates cellular migration [46]. uPA also promotes cell motility via its interaction with uPAR as it regulates the interaction of uPAR to vitronectin and integrins [50] (Figure 2).

**Table 1:** Various model systems studying the involvement of urokinase-type plasminogen activator (uPA) in angiogenesis.

Model	Outcome	Mechanism
uPA and uPAR deficient mice implanted with murine prostate cancer cells	- Substantial decrease in tumor volume. - Significant reduction in angiogenesis. - Tumors showed significantly fewer infiltrating macrophages	Reduced tumor size in uPA and uPAR deficient mice could be due to the reduction of macrophage number [49]
Stable transfection of SNB19 cells with antisense-uPA	- Downregulation of uPA and cellular migration. - Cell cycle arrest in G2/M and interrupted actin cytoskeleton development.	uPa deficiency decreased PI3K and Akt phosphorylation and actin cytoskeleton formation [50]
uPA deficient mice implanted with malignant murine keratinocytes	- Normal tumor angiogenesis. - Increase in tPA activity at the tumor site.	uPA deficiency was recompensed by tPA [51]
MCF-7 cells treated with single-chained uPA (scuPA) and uPA amino-terminal fragment (ATF)	- Increased cell migration	scuPA and uPA ATF induced the Phosphorylation and activation of ERK1/2 [57]

### 2.3. uPAR

uPAR (CD87) is a three-domain glycoprotein (D1, D2, and D3 connected by linker regions) lacking transmembrane and intracellular domains and fastened to the cellular membrane through a GPI anchor [58]. The uPA/vitronectin binding sites are located in D1, but domains 2 and 3 are mandatory for a high-affinity binding [59]. uPAR is expressed on different cell types such as fibroblasts, keratinocytes, trophoblasts, macrophages, monocytes, endothelial, and neural cells. uPAR is 55 kDa in size and was initially known for its role in localizing plasmin degradation to the surface of the cell; subsequent studies, however, unraveled that uPAR stimulates angiogenesis in other proteolysis-independent mechanisms [60,61]. As it is devoid of cytosolic and transmembrane regions, uPAR adopts signaling partners such as members of the integrin subfamilies αv and β1 [60]. In a human epidermoid carcinoma model, the interaction between uPA:uPAR complex and αvβ1 integrin lead to the potent activation of MAPK/ERK pathway in uPAR-rich models. Upon the downregulation of uPAR or the disruption of the uPAR-integrin complex, the MAPK pathway was inhibited, and the cells experienced cell cycle arrest in G_0_/G_1_ phase in vivo [59,62]. Similarly, uPAR acts as a vehicle for the redistribution of αvβ1 integrins to the invasive front of the cell, thereby stimulating EC migration. As VEGFA activates EC through VEGFR-2, it deactivates integrins and activates uPA which in turn forms a complex with uPAR, PAI-1, and αvβ1 integrin. This complex is subsequently internalized through a clathrin-coated pit and uPAR, lipoprotein receptor-related protein (LRP), and integrins are recycled to the edge of the cell [36,60,63]. In vivo, VEGF failed to prompt the internalization of αvβ1 integrin and the subsequent redistribution of integrins in the absence of uPAR, resulting in decreased cellular migration and angiogenesis [64]. uPAR also adopts various additional signaling partners/transmembrane receptors such as focal adhesion kinase (fac), src, and Akt, which are well known for their participation in cancer progression [65]. For example, EC treated with uPAR si-RNA exhibited inhibition in angiogenesis and a decrease in VEGFR2 phosphorylation. This model confirmed the mechanism used by uPAR in inducing VEGFR2 signaling and angiogenic effects. As VEGF binds to VEGFR2, VEGFR2 is dimerized and phosphorylated by tyrosine kinases. uPAR then adopts VEGFR2 as a signaling partner forming a complex held together by β1 integrin. uPAR then uses LRP-1 which induces the endocytosis of this complex and the subsequent activation of the VEGFR2 signaling [66] (Table 2). VEGFR2 phosphorylation thus leads to the activation of various pathways responsible for the proliferation, survival, migration, and vasodilation permeability of EC [19].

Cleavage in the linker region between domains 1 and 2 of uPAR by uPA, plasmin, or MMPs yields two fragments: a fragment consisting of D1 and a fragment consisting of D2 and D3. The latter form exposes an SRSRY sequence and can be cleaved and cleared from the cell surface by phospholipases generating soluble uPAR (suPAR), which in turn stimulates chemotaxis through interacting with G-protein-coupled chemotaxis receptors (GPCR). Phospholipases also cleave full-length uPAR and clear it from the cell surface generating suPAR [58]. In prostate, colorectal and breast cancer patients, high levels of suPAR were detected in plasma and serum [44]. Elevated suPAR levels were also correlated with poor prognosis [67] and low survival rates in cancer patients [68]. In a breast cancer model, it has been found that suPAR induced cellular migration of MCF-7 cells. It has been shown that suPAR induces cellular migration via ERK activation in an epithelial cancer model [59] and in HUVECs grown on tumor condition media [69].

**Table 2:** Various models studying the mechanisms used by uPAR/soluble uPAR in the induction of tumor angiogenesis.

Model	Outcome	Mechanism
HUVEC cells incubated with tumor conditioned media	Enhanced EC invasion and migration	Soluble uPAR from the tumor conditioned media colocalized in membrane lipid rafts on EC and induced ERK/Rac-1 mediated cellular migration and tube formation [69]
- Human and murine EC stimulated with VEGF - Human EC treated with uPAR inhibitory peptides (blocking of uPAR/integrin interaction) - Murine EC retrieved from uPAR deficient mice	- VEGF stimulation enhanced in vivo and in vitro EC migration - Inhibition or loss of uPAR resulted in impaired EC migration in vitro and in vivo	Upon VEGF stimulation, uPAR and integrins interact and are endocytosed via a clathrin-coated vesicle followed by their redistribution to the leading edge of the cell to focus the proteolytic activity of plasmin at the invading side of the cell [64]
- HEp3 carcinoma cells transfected with uPAR antisense mRNA - HEp3 cells transfected with uPAR overexpression vector	- Tumor dormancy and G_0_/G_1_ arrest; decreased association of uPA/uPAR complexes with α5β1 Integrins in uPAR deficient cells - uPAR-rich cells expressed high levels of ERK - uPA/uPAR complexes associated with integrins and exhibited increased tumor migration and progression	High levels of uPAR lead to increased levels of integrins and enhanced adhesion to fibronectin, thus fibronectin-dependent activation of ERK and stimulation of cellular proliferation [62]
HUVEC cells transfected with uPAR small-interfering RNA; subsequent VEGF treatment	- Compromised VEGFR2 signaling - Inhibition of VEGF-induced angiogenesis - Addition of VEGF to HUVECs induced VEGF signaling and angiogenesis	VEGF prompts the interaction of VEGFR2 with uPAR; uPAR then induces the endocytosis of the complex and the activation of VEGFR2 signaling [66]

The expression of uPA and uPAR in tumor cells or neighboring stromal cells is very high, making them both vital prognostic markers for cancer. As seen, reduced tumor invasion was observed in various models using uPAR antagonists, uPAR antisense mRNA, or serine protease inhibitors [51,70]. In fact, an adenovirally delivered uPAR antagonist repressed tumor angiogenesis and growth in vivo [71]. An N-terminal fragment (uPA 1–48) of uPA potentially capable of preventing the binding of uPA to uPAR inhibited EC tubulogenesis in Matrigel. The fragment was capable of suppressing tumor growth and angiogenesis when administered to mice implanted with human glioma cells [21]. Despite the clear significance of the plasminogen activator plasmin system in pathological angiogenesis, their contribution to embryonic and postnatal development seems either trivial or compensated by other proteins since deficiency in tPA, uPA, and uPAR in mice did not affect their embryonic or postnatal development [24]. Plasminogen deficient mice, however, exhibited deficiency in physiological and pathophysiological angiogenesis, highlighting the importance of plasminogen activation for EC tubulogenesis [21]. In physiological angiogenesis, uPAR and uPA play a vital role in regulating immune responses (innate and adaptive) through moderating migration and adhesion of immune cells such as monocytes, neutrophils, and macrophages. In fact, mice deficient in uPAR failed to recruit immune cells such as macrophages to the site of infection [65]. uPA and uPAR are also involved in hematopoietic regulation through the proteolytic activation of ample growth factors and cytokines such as bFGF and interleukin-1 β [72].

### 2.4. Plasminogen/Plasmin

Plasmin is a powerful broad-spectrum serine protease generated from its precursor plasminogen. Plasmin degrades the ECM and liberates major angiogenic growth factors like bFGF and VEGF [26,73]. It is also involved in the ECM remodeling process indirectly via the activation of more than seven pro-MMPs like MMP3, MMP9, MMP12, and MMP13 [43,74]. In an in vitro model, fibrosarcoma cells treated with plasminogen were capable of converting pro-MMP-2 to its mature form; the addition of plasmin inhibitors, however, blocked the activation of MMP-2 [75]. A recent in vivo study subjected wild-type and Plg^−/−^ mice to a stroke and observed their post-stroke recovery. Mice deficient in plasminogen exhibited reduced vessel density compared to the wild type confirming the importance of plasminogen in angiogenesis [76]. In another model, Plg deficient mice transplanted with malignant murine keratinocytes showed a substantial reduction in tumor angiogenesis compared to the wild-type mice [51]. The conversion of VEGF-C and VEGF-D into their active form by plasmin was also shown to promote angiogenesis and lymphangiogenesis [77]. Plasmin itself is regulated by its receptor, which is a p11/Annexin A2 heterodimeric complex. As p11 binds to plasminogen and tPA, it prompts the conversion of plasminogen into plasmin [78]. In vivo, mice deficient in p11 exhibited impaired vascularization [79] and compromised fibrin degradation [80], validating the importance of p11 in plasmin activation. Beside the clear role of plasminogen in cancer, it has been found to play a vital role in the cleavage of cysteine-rich protein 61 (Cyr61), which in turn plays a role in augmenting mesenchymal stem cell-mediated neovascularization in ischemic limbs [81]. Plasmin was also found to convert progalanin released by various types of cancer cells such as MDA-MB231 and BT-549 breast cancer cell lines into its active form galanin. Galanin was shown to induce MMP-9/2 production and promote angiogenesis in vitro and in vivo [82]. Even though plasmin is a very potent proangiogenic molecule, it has also been found to activate certain antiangiogenic molecules. Plasminogen is cleaved by uPA, tPA, MMPs, elastases, and activated plasmin to generate a strong antiangiogenic molecule known as angiostatin [83].

### 2.5. PAI-1

A group of scientists in 1983 were the first to discover PAI-1 produced by cultured bovine aortic ECs and referred to it as a fibrinolytic inhibitor [84]. It is secreted by a number of cells, including adipocytes, platelets, vascular ECs, and hepatocytes [85,86]. PAI-1 is a member of the family of serine protease inhibitors (SERPINs) and is the main inhibitor of uPA and tPA, thereby an inhibitor of pericellular proteolysis and intravascular fibrinolysis, respectively [85]. It is a 45 kDa glycoprotein composed of nine α helices and three β sheets and adopts three conformations based on the state of the reactive center loop (RCL) located on the C-terminus [87]. PAI-1, in its active form, exposes its RCL outwards as an α helix. Upon the binding of uPA/tPA to PAI-1, the active site (p1-p1′) of the RCL domain is cleaved, resulting in the inactivation of plasminogen activators (PAs), incorporation of the N terminal segment of the RCL within the central β sheet, and the formation of an inconvertible complex. It can also exist in a latent conformation in which the entire RCL domain is incorporated within the central β sheet [87,88]. PAI-1 is involved in various physiological processes such as fibrinolysis, ovulation, ECM remodeling, sepsis, and neoplasia [89]. Keratinocytes overexpress PAI-1 during wound healing, emphasizing its important role in tissue repair [90]. An increase in the levels of PAI-1 is associated with several pathological conditions such as cardiovascular and neurodegenerative diseases, cancer, and inflammatory and metabolic disorders [86].

As an inhibitor of uPA, PAI-1 was primarily expected to have anticancer effects; surprisingly, however, high levels of PAI-1 were associated with poor prognosis in various kinds of tumors. According to the Cancer Genome Atlas (TCGA) database, PAI-1 levels are elevated in various types of cancers like stomach adenocarcinoma and head and neck squamous cell carcinoma. Poor clinical outcomes were also associated with high levels of PAI-1 in breast cancer and ovarian serous carcinoma [91]. At the transcriptional level, PAI-1′s expression is stimulated by hypoxia, TGF-β, tumor necrosis factor-α (TNF-α), hormones, cytokines, proteases, and growth factors [92]. Mice sustained in hypoxic environments showed an increase in the levels and the activity of PAI-1 in plasma [93]. It was also shown that several members of the miR-30 family play a direct role in the regulation of PAI-1 by binding to its 3′UTR thus playing a direct role in the regulation of tumor angiogenesis [94].

#### 2.5.1. PAI-1 Promotes Angiogenesis through Interacting with Proteases

PAI-1 protects the ECM against excessive plasmin-mediated proteolysis, thereby maintaining a matrix necessary for ECs to migrate and form capillaries [21,24,95]. In 1878, Billroth confirmed that tumor cells are surrounded by a fibrin thrombus and suggested that fibrin is required for proper tumor proliferation and migration. The role of fibrin in providing tumor and stromal cells with a provisional scaffold for migration has been later elucidated in various studies [36]. A recent study used three-dimensional tumor cells infiltrated with bioengineered blood vessels and embedded in a fibrin-based ECM reported that fibrin stimulated angiogenesis and vascular invasion in vitro [96]. Another study found a correlation between TGF-β, PAI-1, miR-30c, and fibrin-mediated angiogenesis. The study showed that TGF-β causes a decrease in miR-30 expression resulting in the upregulation of PAI-1. PAI-1 subsequently inhibits uPA and the consequent formation of plasmin yielding finally in the accumulation of fibrin and the stimulation of angiogenesis [94] (Table 3). We previously demonstrated that we could restore tumor angiogenesis in mice deficient for PAI-1 and transplanted with malignant murine keratinocytes using mutated recombinant PAI-1 at the vitronectin binding site. However, mutated PAI-1 at the anti-proteolytic site was inefficient in restoring tumor angiogenesis. The same delayed effect was seen in plasminogen deficient mice, thereby highlighting the importance of plasmin-mediated proteolysis in angiogenesis. Restoration of the levels of PAI-1 in the circulation was able to restore angiogenesis and promote tumor growth in PAI-1 deficient mice [51].

#### 2.5.2. PAI-1 Promotes Angiogenesis through Binding to Vitronectin

In plasma, PAI-1 is bound to the matrix protein vitronectin, which stabilizes PAI-1 in its active configuration and extends its half-life >10 times [85]. On the N-terminus of vitronectin exists a somatomedin B (SMB) domain of 44 amino acids that acts as a binding site for PAI-1 and uPAR; contiguous to it lies an RGD domain which is the binding site for integrins such as α_v_β_1_, α_v_β_3_, and α_v_β_5_ [97,98]. PAI-1 interacts with a variety of molecules other than PAs such as vitronectin, heparin, and LRP to mediate cell migration [99]. PAI-1 and uPAR bind to vitronectin through the SMB domain, which, except for the binding affinity of PAI-1, are noticeably higher than the affinity of uPAR to the same domain, thereby inhibiting uPAR/integrin-dependent cell attachment to vitronectin [24]. Cells also detach from other ECM proteins such as fibronectin, laminin, and collagen. The cryptic receptor-binding site (CRB) of PAI-1 is hence exposed, increasing its affinity to the endocytic receptor LRP and decreasing its affinity to vitronectin resulting in the endocytosis of the uPA:uPAR:PAI-1:LRP complex. Lysosomes target uPA and PAI-1 for degradation and the cell recycles LRP and uPAR to the leading edge of the cell to form new connections with the ECM [99,100,101]. To attain an environment favorable for cellular migration, a balance between uPA and PAI-1 must be sustained. In fact, an increase in the levels of PAI-1 has been linked to poor clinical outcomes in many types of cancer [89]. Studies confirmed that ECs move from vitronectin-rich vascularized areas to fibronectin-rich non-vascularized areas with the help of PAI-1 [21]. Inert ECs do not normally express uPA, uPAR, and PAI-1 but do so when stimulated by angiogenic factors and/or hypoxia [10,21]. Using human brain endothelial cells, PAI-1 was found to promote the migration of EC from their perivascular space rich in vitronectin towards the tumor tissue rich in fibronectin [102]. In another model system, PAI-1 hindered the adhesion of human fibrosarcoma cells to vitronectin and stimulated the migration of the cells from vitronectin to Collagen type IV. The use of antibodies against α_v_β_5_ integrins prompted the same response seen when the cells were treated with recombinant PAI-1, confirming that α_v_β_5_ integrins bind to vitronectin and PAI-1 competes with its binding to vitronectin stimulating cellular migration [103].

#### 2.5.3. PAI-1 Promotes Angiogenesis through Inhibition of Apoptosis

**Figure 3:**
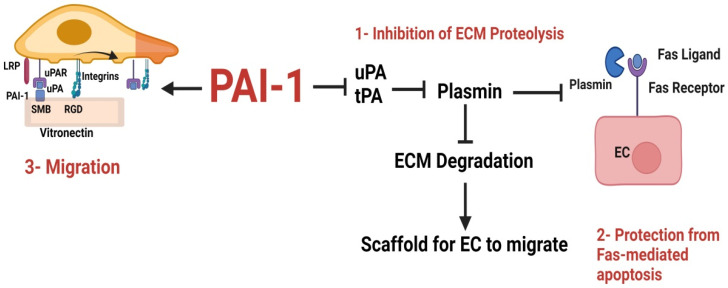
PAI-1 Promotes angiogenesis by three known methods. (1) PAI-1 inhibits uPA and tPA from activating plasmin, thereby protecting the ECM from plasmin-mediated proteolysis and providing EC with a scaffold to migrate and proliferate. (2) PAI-1 inhibits plasmin activation, thereby preventing the release of the Fas ligand as soluble fas. (3) PAI-1 promotes EC migration through interacting with vitronectin.

We have previously shown that PAI-1 protects EC from fasL-mediated apoptosis and confirmed that in the absence of PAI-1, apoptosis is enhanced via plasmin-mediated Fas-L cleavage at Arg^144^-Lys^145^ [104]. Similarly, another study used tumor cell lines stably transfected with hairpin PAI-1 si-RNA or a PAI-1 inhibitor and showed that there was a significant increase in spontaneous apoptosis in vitro. The subsequent treatment of PAI-1 deficient tumor cells with recombinant PAI-1 or Fas/FasL antibodies abolished apoptosis, confirming that PAI-1 protects EC from Fas/FasL-mediated apoptosis. PAI-1 knockout or immunodeficient mice transplanted with tumor cells also displayed reduced tumor growth and angiogenesis in vivo [105] (Figure 3).

**Table 3:** Model systems studying the mechanisms used by PAI-1 to induce tumor angiogenesis.

Model	Outcome	Mechanism
- Mice with a postnatal deletion of transforming growth factor-β (TGF-β) - Treatment of wild type mice with nanoparticles carrying miR-30 antagomiR	- TGF-β deletion resulted in inhibition in fibrin-mediated angiogenesis - The nanoparticle treatment instigated tumor growth, angiogenesis, and fibrin accumulation	In the absence of TGF-β, an increase in miR-30′s expression causes a decrease in PAI-1′s expression and the subsequent accumulation of plasmin which instigates the degradation of fibrin and the inhibition of angiogenesis [94]
- Mice with PAI-1 deficiency implanted with human neuroblastoma cells - HBMEC cells transfected with PAI-1 siRNA - HBMEC cells deficient in PAI-1 treated with anti-Fas antibodies	- Reduction in tumor size and vascularization - Elevated number of apoptotic EC - Increase in spontaneous apoptosis - Inhibition of apoptosis	Knockdown of PAI-1 enhances plasmin activity which cleaves Fas ligand and releases it as a soluble 21.5 kDa soluble protein with proapoptotic properties [105]
Adenovirus-mediated gene transfer of mutated PAI-1 (PAI-1 deficient in vitronectin binding or in plasminogen activators inhibition) to PAI-1 deficient mice transplanted with malignant murine keratinocytes	Restoration of tumor angiogenesis with recombinant PAI-1 mutated at the vitronectin interaction site	PAI-1 induces tumor angiogenesis and invasion through its interaction with proteases, not vitronectin [51]

PAI-1^−/−^ mice implanted with malignant murine keratinocytes did not display tumor invasion or vascularization compared to wild-type mice. Adenovirus-mediated gene transfer of PAI-1 to the PAI-1^−/−^ mice restored tumor invasion and angiogenesis [106]. Transient transfection of MDA-MB-231 and HUVECs cell lines with RNA aptamers capable of inhibiting PAI-1 displayed a significant decrease in tumor cell migration and invasion [107]. We have previously shown that 16K prolactin forms a relatively stable complex with PAI-1, thereby inhibiting it in the pathology of thrombosis [108]. However, the inhibitory effect of 16K prolactin is still under investigation to understand the complex interaction between the two molecules. High levels of PAI-1 are not only limited to cancer but are also associated with other nontumoral pathologies such as diabetic retinopathy or age-related macular degeneration (AMD). Mice deficient in PAI-1 exhibited suspended choroidal angiogenesis in an AMD model [109].

Using malignant keratinocytes orthotopic tumor model, wild-type, PAI-1-deficient, and PAI-1-overexpressed mice were transplanted with PVDA cells overexpressing PAI-1. Both PAI-1-overexpressed mice and PAI-1-deficient mice showed regression in tumor angiogenesis. It was postulated that PAI-1, at normal physiologic concentrations, promotes angiogenesis; at supraphysiologic concentrations, however, PAI-1 inhibits tumor invasion and angiogenesis. The cellular source of PAI-1, whether of tumor or stromal origin, is a vital factor in tumor angiogenesis since high levels of PAI-1 from the tumor keratinocyte cells could not compensate for the deficiency of PAI-1 in the host cells [110]. Paradoxically, an in vivo study showed no significant differences in primary tumor growth and pulmonary metastasis between mice overexpressing PAI-1 and mice deficient in PAI-1 [111]. Similarly, PAI-1 stably transfected human prostate carcinoma cells displayed a diminution in tumor growth, angiogenesis, and metastasis [112]. These contradictory results may be due to the different concentrations of PAI-1 expressed by tumor cells, tumor models used, and to the various roles played by PAI-1 [53].

#### 2.5.4. PAI-1 in Vessel Attrition

In a murine model of accelerated aging, mice with a mutation in the *klotho* gene showed an increase in the levels of PAI-1 antigen in plasma, a decrease in the levels of uPA mRNA, and an increase in PAI-1 mRNA in various tissues like the kidneys, heart, and blood vessels. The increase was age-related and was associated with an increase in fibrin deposition in the kidneys suggesting that PAI-1 plays a direct role in tissue/organ damage via the impairment of microcirculation and induction of micro-thrombosis [113]. In fact, high levels of PAI-1 in the elderly cause the accumulation of fibrin and ECM components such as collagen in tissues leading to tissue fibrosis [114]. Similarly, mice with combined deficiencies in *klotho* and *SERPINE1* genes exhibited prolonged lifespans and normal organ structure and function [115]. Another study revealed a loss-of-function mutation in the *SERPINE1* gene, which was found to protect against aging [116]. Altogether, the previous data confirms that PAI-1 levels increase with age and that PAI-1 plays a vital role in aging via blocking fibrin degradation and supporting the accumulation of ECM components in the tissues and vessels, leading to vessel attrition and tissue aging. Recent studies indeed confirmed that vascular attrition is one of the hallmarks of aging [117] and that a decrease in blood vessel density could be associated with organ aging [118].

### 2.6. PAI-2

PAI-2 is a potent uPA inhibitor that exists in two forms: a 47 kDa nonglycosylated intracellular form and a secreted 60 kDa glycosylated form, but exists mainly in the prior form. The expression of the PAI-2 *SERPINB2* gene is induced by inflammatory mediators such as TNF-α and lipopolysaccharides or by viral and bacterial infections [119,120,121]. It is expressed constitutively by various cell types like keratinocytes and syncytial trophoblasts and is also expressed as a result of inflammation or injury by various cell types, including monocytes, macrophages, EC, and fibroblasts, highlighting its important role in mediating wound healing and inflammation [122]. PAI-2 levels are also elevated in trophoblasts emphasizing its vital role in embryogenesis; reduced plasma levels of PAI-2 in human embryos were associated with preeclampsia and intrauterine growth delay [121]. However, in vivo experiments on mice showed that mice deficient in PAI-2 exhibited normal development and survival [120]. Unlike PAI-1, elevated levels of PAI-2 in cancer are linked with reduced cancer progression and metastasis due to its inability to bind to vitronectin. Similarly, PAI-2 downgrades vitronectin-dependent cellular movement via competing with PAI-1 bound to vitronectin for uPA binding [121]. A recent study using PAI-2 deficient mice orthotopically injected with melanoma or lung tumor cells confirmed that the loss of PAI-2 increased the predisposition to metastasis and tumorigenesis in vivo [120]. On the other hand, studies have demonstrated that high levels of PAI-2 were associated with increased metastasis and lower survival percentages in patients with breast cancer via the upregulation of PAI-2 by microRNA-200c [123,124]. Likewise, a recent study used PAI-1 knockout mice and confirmed that PAI-2 compensated for the loss of PAI-1 and supported bladder cancer progression in vivo [125]. Unlike PAI-1, PAI-2 is not extensively studied and the role it plays in angiogenesis is not fully understood.

## 3. Antiangiogenic Therapy: Targeting Serine Proteases

Targeting angiogenesis is one of the major therapeutic strategies in controlling tumor growth and other pathological conditions characterized by neovascularization. As serine proteases play vital roles in homeostasis, the clinical use of synthetic serine protease inhibitors has been limited substantially. Nonetheless, the drug upamostat (RHB-107) is an oral uPA inhibitor that was granted FDA approval in 2017 for the adjuvant treatment of pancreatic cancer and is currently in phase II clinal trials [43]. Upamostat showed adequate slight toxicity and lower mortality rates in pancreatic cancer patients in clinical trials [126]. Advanced head and neck squamous cell carcinoma (HNSCC) patients administered with the active metabolite of the drug upamostat WX-UK1 or the oral pro-drug WX-671 showed no differences in the plasma pharmacokinetics compared to the healthy group and only experienced mild symptoms like diarrhea [127]. In vitro, a 50% reduction in tumor invasion and migration and a significant decrease in the ability of carcinoma spheroids to migrate towards fibroblast spheroids was achieved in various carcinomas after the treatment with WX-UK1 [128]. In vivo, WX-UK1 inhibited the proteolytic activity of uPA and plasmin in mice and reduced tumor growth and metastasis [129], perhaps by inhibiting plasmin-mediated ECM proteolysis and MMP activation. Due to the hydrophobicity of WX-UK1, shortly after the intravenous administration of the molecule, it localizes in tissues and leaves the bloodstream in a matter of minutes, thereby not influencing coagulation factors in the blood such as thrombin and Factor Xa [129]. Despite the success seen in clinical trials and the absence of any coagulation-related diseases, Upamostat showed low specificity for uPA and an inhibition potential of homologous proteases such as coagulation factors. Thus, studies are more directed towards finding drugs with higher specificity and efficiency against their target in order to maintain homeostasis [43]. Another synthetic uPA inhibitor known as Amiloride was also capable of reducing angiogenesis in a CAM assay [130] and decreasing the migration and invasion of HeLa cells in vitro [131]. The use of Amiloride in cancer therapy, however, is limited due to the diuretic effects of this drug which can cause adverse side effects in cancer patients such as dehydration. For the potential use of this drug in clinics, scientists have found analogues for Amiloride with higher selectivity and enhanced activity against uPA. These derivatives lacked the diuretic effects seen in their original counterparts and displayed anti-metastatic effects in vivo [132]. Tranexamic acid (TXA) is another pharmaceutical molecule that is a lysine analogue that blocks lysine binding sites on Plg, thereby inhibiting Plg activation and fibrin hydrolysis [133]. Mice orthotopically injected with lung carcinoma cells transfected with progalanin-siRNA and then treated intraperitoneally with TXA showed a significant decrease in MMPs activation and in angiogenesis in vivo. TXA inhibited plasmin, thereby inhibiting progalanin activation and the subsequent activation of MMPs [82]. Recently, TXA was found to significantly impede HUVEC cells tube formation and angiogenesis in vitro in the pathology of melasma, a skin condition characterized by increased dermal vascularity, via the inhibition of VEGFRs activation [134]. Various studies on melasma patients administered with TXA demonstrated that oral TXA is efficient in the treatment of this skin condition and a safe therapeutic option with mild side effects. As this drug targets plasmin, a protease implicated in fibrinolysis, the risk of thromboembolism might be a matter of concern; to date, however, TXA showed no increased risk of thrombosis in patients [135]. Up to date, most of the drugs directed against uPAR target uPAR itself or the interaction of uPAR with other proteins such as uPA, vitronectin, integrins, and receptors such as formyl peptide receptor (FPR). Such inhibitory molecules include peptides, small-molecule composites, recombinant proteins, and monoclonal antibodies [136]. Small synthetic peptides known as AE120 and AE105 were found to interfere with uPA:uPAR interaction and inhibit invasion of squamous carcinoma cells in the in vivo CAM assay [137]. Another molecule, UPARANT, is a uPAR-derived peptide (uPAR Ser^88^-Tyr^92^) that targets the uPAR:FPRs interaction and competes with FPR ligands for the FPR binding site, thereby inhibiting the activation of downstream signaling pathways responsible for the instigation of angiogenesis. By the aforementioned mechanism, UPARANT was found to abrogate laser-induced CNV in vivo, making UPARANT an attractive therapeutic molecule for AMD. In vivo, mice administered with UPARANT for the treatment of diabetic retinopathy showed promising results in preventing the onset of retinal neovascularization without causing harmful side effects [138]. PAI-1 inhibitors currently under investigation target PAI-1 itself or the interactions between PAI-1 and plasminogen activators or other binding partners of PAI-1 such as LRP1. Another approach is to catalyze the conversion of active PAI-1 into an inert or inactive conformation [139]. A carboxylic-acid-derived small molecule termed TM5007 was able to inhibit PAI-1 and coagulation in vivo via binding to the strand 4 position (s4A) in PAI-1 β-sheet, thereby inhibiting PAI-1 activity [140]. We have previously demonstrated that a 16KDa fragment of the hormone prolactin inhibited PAI-1 and coagulation in vivo [108]. Likewise, small peptides derived from growth hormone, placental lactogen, and prolactin were found to be efficient in the treatment of thrombosis-related diseases. In conclusion, serine proteases, specifically the plasminogen activator plasmin system, play a vital role in pathological angiogenesis and developing pharmaceutical molecules targeting them could be an efficient approach to control cancer and diseases characterized by neovascularization.

## 4. Concluding Remarks and Perspectives

Ample clinical and experimental data support the involvement of members of the plasminogen activator plasmin system in pathological angiogenesis, especially in cancer. Discrepancies seen in some studies could be explained by the complexity of the system as these proteases act on multiple substrates and extend their effects on many molecules [21]. Trying to further understand the cellular and molecular mechanisms of angiogenesis and the involvement of these molecules in its pathogenesis is vital for the development of therapeutic synthetic molecules targeting serine proteases in various pathologies characterized by neoangiogenesis [24]. The use of modulators of the plasminogen system is a double-edged sword since the body works as one entity and meddling with this state of homeostasis might render the body in a state of a homeostatic imbalance. Blocking plasmin or tpa for example, might show antiangiogenic effects but still might render the body incapable of performing normal physiological functions that depend on plasmin such as fibrinolysis. Similarly, these modulators might have effects on other molecules besides their primary target, thereby affecting other untargeted processes. Thus, we still need to improve the selectivity, efficacy, and safety of drugs for their potential use in clinics.

## Data Availability

Not applicable.
